# The “Environment” for Autism Research: Signs of Improvement?

**DOI:** 10.1289/ehp.12107

**Published:** 2008-10

**Authors:** Cindy P. Lawler

**Affiliations:** National Institute of Environmental Health Sciences, Division of Extramural Research and Training, Research Triangle Park, NC, E-mail: lawler@niehs.nih.gov

Once thought to be rare, autism spectrum disorders (ASD) have gained increasing public attention as prevalence studies have revealed sharp increases over the past two decades. ASD now represents the second most common category of neurodevelopmental disorders. The prevalence data have been instrumental in mobilizing parents to act on behalf of their affected family members for better services and treatments and for greater research investments aimed at understanding what causes these disorders and how they can be prevented.

Increased funding for ASD research has led to visible progress in many areas, although the causes for the sharp increase in prevalence remain unresolved. Changes in awareness, diagnostic practices, and availability of services can account for some of the increase, yet a separate role for increased exposure to one or more environmental agents also has been suggested.

The increases in prevalence data also helped fuel speculation regarding a link between the mercury-based vaccine preservative thimerosal and ASD. Many methodologically sound epidemiologic studies have failed to support a causal relationship. Concerns persist for some parents, however, and current time trends remain under scrutiny. The possibility of a small subgroup of children with a unique vulnerability to vaccines or vaccine components has been raised and is more difficult to address.

In light of the persistent public attention to ASD prevalence and to vaccines as potential risk factors, it is surprising that an appreciation for the larger fundamental question of how nongenetic/environmental factors may contribute to ASD has grown slowly. This is unfortunate, because a diverse spectrum of environmental factors merit consideration—from pesticides and other agrichemicals to pharmaceuticals, nutrition, and lifestyle. The full explanation for the relative lack of attention to environmental hypotheses is complex. Contributing influences are likely to include the contemporary scientific focus on genetic etiologies of ASD, the possible reluctance of investigators to enter an arena that included the controversial issue of vaccines as risk factors, the lack of strong etiologic clues to pursue, and the need to build new investigative teams and infrastructure that can support sustained research in this nascent area. A recent Institute of Medicine meeting on autism and the environment (Autism and the Environment: Challenges and Opportunities for Research, Washington, DC, 16–17 April 2007) highlighted the continuing challenges and opportunities for research in this area (Altevogt et al. 2008).

Despite the relatively tepid scientific interest in the environment and autism over the past 8 years, there have been signs of progress. A U.S. surveillance network established by the Centers for Disease Control and Prevention (CDC) is now in place to aid detection and interpretation of future trends (Rice et al. 2007). In 2001, a Center for Children’s Environmental Health and Disease Prevention whose focus is on ASD was established at the University of California-Davis with support from the National Institute of Environmental Health Sciences and U.S. Environmental Protection Agency. The Center brought new investigators into the field of autism and environmental health sciences and incorporated methods of community engagement that built trust between researchers and parent advocates; this, in turn, enriched the science being conducted and enhanced the ability to disseminate scientific results produced by the studies. Interdisciplinary collaborations among investigators at the Center make it possible to tackle questions using multiple approaches, from human studies to animal and cellular models.

One key component of the University of California-Davis Center has been the CHARGE (Childhood Autism Risks from Genetics and the Environment) study, the first large scale population-based case–control epidemiology study of environmental and genetic risk factors for autism. Various environmental risk factors are being explored, from metals and pesticides to medications and infections (Hertz-Picciotto et al. 2006). The nascent research efforts begun in 2001 have matured and expanded; recent findings of note support a role for immune dysregulation in ASD.

More recently, other large U.S. epidemiology studies have been initiated that can help address the relationship between ASD and environmental factors, including the CDC-sponsored SEED (Studies to Explore Early Development) Study (CDC 2006).

One of the recently awarded National Institutes of Health (NIH) Autism Centers for Excellence (ACE) is a multisite collaborative study led by investigators at Drexel University focused on identifying environmental risks, biomarkers, genetic susceptibility, and gene–environment interplay (NIH 2008). The EARLI study (Early Autism Risk Longitudinal Investigation) will create an enriched risk birth cohort. Mothers with one child with ASD will be enrolled at the start of a subsequent pregnancy, and a variety of data, including data related to environmental exposures, will be collected pre- and postnatally to address key questions regarding etiology and natural history of ASD.

Perhaps the most promising indicator of future progress is evident in the recent activities of the Interagency Autism Coordinating Committee (IACC). The IACC was reestablished as part of the 2006 Combating Autism Act and charged with developing and annually updating a Strategic Plan for Autism Research (IACC 2007) to guide federal efforts and encourage public–private partnerships. The process of plan development has included gathering broad input from various stakeholders and evaluating current research investments to identify strengths, weaknesses, and opportunities. There is general agreement that the role of environment in ASD risk has been neglected and merits additional emphasis. The draft version of the plan includes several objectives that address environmental aspects of autism.

Finally, it is important to recognize that advances in understanding environmental influences in ASD will rely on developments in other areas. The NIH Genes, Environment and Health Initiative (NIH 2007) should provide new tools and approaches for addressing joint effects of genetic and environmental influences in complex disorders. The growing field of epigenomics suggests new ways for the environment to induce heritable changes in gene regulation without altering gene sequence. Advances in stem cell biology that enable reprogramming of fibroblasts from individuals with complex diseases could be used to probe susceptibility to environmental exposures during the earliest stages of cell development. Likewise, new developments in ASD genetics, such as copy number variation, offer novel opportunities to consider how environmental agents may influence genomic stability.

In summary, the “environment” for this area of research has improved, albeit slowly. Incorporation of specific objectives targeting environmental risk factors in the IACC Strategic Plan, together with clues emerging from ongoing studies and an increased recognition of the potential for identification of preventable risk factors, should help to create a new sense of urgency to address environmental hypotheses in ASD.

## Figures and Tables

**Figure:**
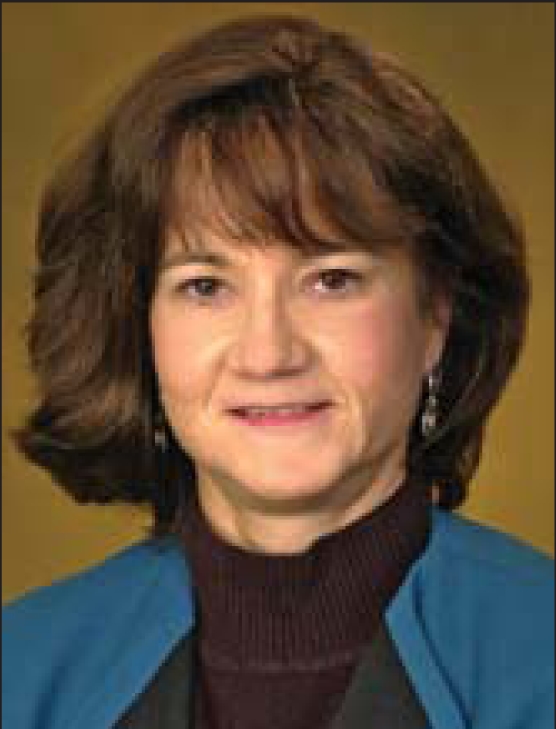
Cindy P. Lawler

## References

[b1-ehp-116-a416] Altevogt BM, Hanson SL, Leshner AI (2008). Autism and the environment: challenges and opportunities for research. Pediatrics.

[b2-ehp-116-a416] CDC (Centers for Disease Control and Prevention) (2006). Studies to Explore Early Development (SEED).

[b3-ehp-116-a416] Combating Autism Act. 2006. Public Law 109-416.

[b4-ehp-116-a416] Hertz-Picciotto I, Croen LA, Hansen R, Jones CR, van de Water J, Pessah IN (2006). The CHARGE study: an epidemiologic investigation of genetic and environmental factors contributing to autism. Environ Health Perspect.

[b5-ehp-116-a416] IACC (2007). Interagency Autism Coordinating Committee (IACC) Strategic Plan for Autism Spectrum Disorder Research.

[b6-ehp-116-a416] NIH (National Institutes of Health) (2007). The Genes, Environment and Health Initiative (GEI).

[b7-ehp-116-a416] NIH (National Institutes of Health) (2008). Newly Awarded Autism Centers of Excellence to Further Autism Research.

[b8-ehp-116-a416] Rice CE, Baio J, Van Naarden Braun K, Doernberg N, Meaney FJ, Kirby RS (2007). A public health collaboration for the surveillance of autism spectrum disorders. Paediatr Perinat Epidemiol.

